# Some Records from Insurance Practice

**Published:** 1920-10-02

**Authors:** 


### THE STATISTICS OF HEART DISEASE.

### Some Records from Insurance Practice.

WITH reference to its increasing incidence, statistical records of heart disease are undoubtedly among the least satisfactory which the medical world has before it, and although one cannot expect to draw exact parallels between countries of such essentially different economic conditions as those which border on the Eastern and Western Atlantic respectively, yet there is no little interest to be drawn from the report of Dr. F. L. Hoffman, a Vice-President and statistician of the Prudential Insurance Company of the U.S.A.

This author has provided a number of valuable papers towards what may be called true preventive medicine, and the fact that he takes the viewpoint of insurance rather than clinical practice gives his writings a very definite claim to consideration in this country at a time when we need every atom of available evidence if a really efficient health campaign is to be carried out.

One must sympathise with his initial plea that the terminology in cardiac conditions, if it does not leave much to be desired, is at least extraordinarily resistant to definite analysis. The statistician's difficulty is aptly illustrated by the late Dr. Charles A. Mercier when he observed, "The fatal manifestation of a disease is, I surmise, what the Registrar-General means by a terminal condition or mode of death; but as he gives no indication whatever as to what he does mean, this can be no more than a surmise. In such cases the disease may appropriately be called the principal cause of death and the manifestations, the precipitating or subordinate cause of death." And still we meet with undiminished frequency the all-embracing phrase, death from cardiac failure.

Obviously a minute proportion of such cases are really deaths from cardiac disease. How many of these cases would have shown clinical signs of heart disease during life? In practice, whatever classification is made, wherever the line is drawn between the normal and the deficient cardiac state, the guiding principle must, in the words Sir James Mackenzie, be not only the recognition of the classical signs of disease as such, but also appreciation of the fitness of any man's heart to perform, and to continue to perform, the daily task which it is likely to encounter. And it is with due recognition of this need and the more obvious call for a "kind of knowledge which nowhere exists" that Dr. Hoffman set about collecting his records of the incidence of heart disease, or more accurately, the percentage occurrence of relatively deficient cardiac power among the manhood of America.

In both our own country and his, the relative frequency of heart disease among men of military age has become automatically known—or partially known—through the war-time records and to a lesser extent by years of insurance practice. In the latter, however, a certain degree of selection occurs, in that the more obvious cases of cardiac impairment do not present themselves for examination, and in both the methods of examination have hardly been sufficiently perfected to justify rejection of a fair proportion of cases on the basis of mere murmurs, the true nature of which is not understood at the present time. Also, Dr. Hoffman explains, in the experience of the Prudential Company during the period 1915-18, the rejections on account of heart impairments have been 24.4 per thousand examined, but the fact must not be overlooked that the practice of different companies varies. Nevertheless medico-actuarial experience gives some valuable information, and essentially includes an aspect of cardiac disease which can never be fully represented by voluntary or other hospital's records, and it may be well, therefore, to examine some figures obtained on the broader lines.

Table I. clearly indicates the trend of recorded mortality figures; although there must be a small percentage of error from death-certificate inaccuracy.

*Mortality from Heart Diseases, U.S. Registration Area,*
1900-1918.

**Death-Rates per Hundred Thousand of Population.**

| Year | All Forms of Heart Disease | Peri-carditis | Acute Endo-carditis | Organic Heart Disease | Angina Pectoris |
|---|---|---|---|---|---|
| 1900 | 131.9 | 2.6 | 11.9 | 111.1 | 6.4 |
| 1905 | 152.1 | 1.7 | 12.5 | 131.2 | 6.7 |
| 1910 | 158.8 | 1.2 | 8.9 | 141.5 | 7.2 |
| 1915 | 165.1 | 1.1 | 9.1 | 147.1 | 7.7 |
| 1916 | 168.0 | 1.0 | 9.3 | 160.1 | 7.6 |
| 1917 | 170.9 | 1.1 | 8.9 | 153.1 | 7.9 |
| 1918 | 169.0 | 1.1 | 8.2 | 152.3 | 7.4 |

The rates of pericarditis and acute endocarditis have decidedly decreased. But in contrast all forms of heart disease have increased from 131.9 per 100,000 in 1900 to 169.0 in 1918. It is true that within recent years increasingly careful investigation of history, etc., may have accounted for some *apparent* increase, yet it is unlikely to explain it all.

Next, if the age incidence be considered, it is seen that if we take the proportionate mortality from different causes, without reference to sex, heart diseases as a group constitute 18.8 per cent. of the mortality from all causes at the ages of forty and over. Equally important is the fact that cardiac mortality at ages of forty and over constitute 85.8 per cent. of the mortality from heart diseases at *all* ages; in other words, the condition is essentially prominent in advanced adult life. This fact in itself simply places the cardiac state parallel with those in cancer, tuberculosis, pneumonia, and several others, but Dr. Hoffman gives a striking table proving that considerable importance is to be attached to the increase of heart incidence as a whole, cancer alone falling in the same category.

The comparative figures concerning the white

**The Statistics of Heart Disease**—(*continued*).

and black races are not of so much interest to this country, and in any case they deal largely with the half-explored field of race pathology. But generally they seem to sustain the point of view that the negro is more liable to this group of diseases, particularly acute endocarditis, than is the white man.

Of more vital interest to our readers is the author's statistical disproof of the alleged decrease of heart affections in the British Isles. According to his finding the facts are quite contrary as regards England and Wales, and the following table shows that heart diseases apparently increased in this country from 147.9 per hundred thousand in 1900 to 172.1 in 1915. Equally suggestive is the apparent increase in deaths from apoplexy and diseases of the blood-vessels from 87.1 per hundred thousand in 1900 to 118.0 in 1915.

With Dr. Hoffman, the reader will realise that international vital statistics are to be used for comparative purposes with extreme caution, and that few are in a position to make sure of the parallels drawn, unless they have made personal inquiry in the various countries concerned. There is, indeed, an unquestionable difference of viewpoint in the medical worlds of England and America. But such statistics may be relied upon in a general way if drawn from identical methods of investigation, and in this case well-established insurance practice is the basis of both.

*Mortality in England and Wales from Specified Causes of Death.*

**Crude Death-Rates per Hundred Thousand of Population.**

| Year | All Causes* | Cancer | Pulmonary Tuberculosis | Heart Diseases | Apoplexy and Diseases of the Blood Vessels | Pneumonia, All Forms | Nephritis and Bright's Dis. | Violence | Old Age |
|---|---|---|---|---|---|---|---|---|---|
| 1900 | 18 2 | 82.9 | 133.3 | 147.9 | 87.1 | 137.4 | 40.1 | 62.4 | 93.3 |
| 1905 | 15.3 | 88.9 | 114.6 | 142.3 | 83.9 | 130.6 | 37.6 | 57.1 | 92.8 |
| 1910 | 13.5 | 96.7 | 101.5 | 136.1 | 88.6 | 111.0 | 38.9 | 52.2 | 89.0 |
| 1915† | 15.7 | 112.1 | 113.6 | 172.1 | 118.0 | 135.9 | 45.5 | 56.4 | 89.7 |
| 1917† | 14.4 | 121.0 | 122.8 | 166.6 | 121 9 | 114.4 | 41.9 | 54.9 | 88.8 |
| **Number of Deaths to every 100 in 1900.** | | | | | | | | | |
| 1905 | 84 | 107 | 86 | 96 | 96 | 95 | 94 | 92 | 94 |
| 1910 | 74 | 117 | 76 | 92 | 102 | 81 | 97 | 84 | 91 |
| 1915 | 86 | 135 | 85 | 116 | 135 | 99 | 113 | 90 | 91 |
| 1917 | 79 | 146 | 92 | 113 | 140 | 83 | 104 | 88 | 90 |

* Rate per thousand.

† Based on civil population only. Years 1915 and 1917 are not strictly comparable with previous years or with United States data.

